# The emergence of carbapenemase-producing *Enterobacterales* in hospitals: a major challenge for a debilitated healthcare system in Lebanon

**DOI:** 10.3389/fpubh.2023.1290912

**Published:** 2023-11-20

**Authors:** Dina Daaboul, Saoussen Oueslati, Mariam Rima, Issmat I. Kassem, Hassan Mallat, Aurélien Birer, Delphine Girlich, Monzer Hamze, Fouad Dabboussi, Marwan Osman, Thierry Naas

**Affiliations:** ^1^Faculty of Medicine, Team "Resist" UMR1184, "Immunology of Viral, Auto-Immune, Hematological and Bacterial Diseases (IMVA-HB)," INSERM, Université Paris-Saclay, CEA, Health and Therapeutic Innovation (HEALTHI), Le Kremlin-Bicêtre, France; ^2^Laboratoire Microbiologie Santé et Environment (LMSE), Doctoral School of Sciences and Technology, Faculty of Public Health, Lebanese University, Tripoli, Lebanon; ^3^Bacteriology-Hygiene Unit, Assistance Publique-Hôpitaux de Paris, AP-HP Paris-Saclay, Bicêtre Hospital Le Kremlin-Bicêtre, Paris, France; ^4^Center for Food Safety and Department of Food Science and Technology, University of Georgia, Griffin, GA, United States; ^5^French National Reference Center for Antibiotic Resistance, Clermont-Ferrand, France; ^6^Cornell Atkinson Center for Sustainability, Cornell University, Ithaca, NY, United States; ^7^Department of Public and Ecosystem Health, College of Veterinary Medicine, Cornell University, Ithaca, NY, United States; ^8^Department of Neurosurgery, Yale University School of Medicine, New Haven, CT, United States

**Keywords:** Enterobacterales, antimicrobial resistance, one health, ESBL, carbapenemases

## Abstract

**Background:**

Carbapenem- and extended-spectrum cephalosporin-resistant *Enterobacterales* (CR-E and ESCR-E, respectively) are increasingly isolated worldwide. Information about these bacteria is sporadic in Lebanon and generally relies on conventional diagnostic methods, which is detrimental for a country that is struggling with an unprecedented economic crisis and a collapsing public health system. Here, CR-E isolates from different Lebanese hospitals were characterized.

**Materials and methods:**

Non-duplicate clinical ESCR-E or CR-E isolates (*N* = 188) were collected from three hospitals from June 2019 to December 2020. Isolates were identified by MALDI-TOF, and their antibiotic susceptibility by Kirby-Bauer disk diffusion assay. CR-E isolates (*n* = 33/188) were further analyzed using Illumina-based WGS to identify resistome, MLST, and plasmid types. Additionally, the genetic relatedness of the CR-E isolates was evaluated using an Infrared Biotyper system and compared to WGS.

**Results:**

Using the Kirby-Bauer disk diffusion assay, only 90 isolates out of the 188 isolates that were collected based on their initial routine susceptibility profile by the three participating hospitals could be confirmed as ESCR-E or CR-E isolates and were included in this study. This collection comprised *E. coli* (*n* = 70; 77.8%), *K. pneumoniae* (*n* = 13; 14.4%), *Enterobacter* spp. (*n* = 6; 6.7%), and *Proteus mirabilis* (*n* = 1; 1.1%). While 57 were only ESBL producers the remaining 33 isolates (i.e., 26 *E. coli*, five *K. pneumoniae*, one *E. cloacae*, and one *Enterobacter hormaechei*) were resistant to at least one carbapenem, of which 20 were also ESBL-producers. Among the 33 CR-E, five different carbapenemase determinants were identified: *bla*_NDM-5_ (14/33), *bla*_OXA-244_ (10/33), *bla*_OXA-48_ (5/33), *bla*_NDM-1_ (3/33), and *bla*_OXA-181_ (1/33) genes. Notably, 20 CR-E isolates were also ESBL-producers. The analysis of the genetic relatedness revealed a substantial genetic diversity among CR-E isolates, suggesting evolution and transmission from various sources.

**Conclusion:**

This study highlighted the emergence and broad dissemination of *bla*_NDM-5_ and *bla*_OXA-244_ genes in Lebanese clinical settings. The weak AMR awareness in the Lebanese community and the ongoing economic and healthcare challenges have spurred self-medication practices. Our findings highlight an urgent need for transformative approaches to combat antimicrobial resistance in both community and hospital settings.

## 1 Introduction

The order Enterobacterales includes the most common human bacterial pathogens responsible for community- and healthcare-associated infections. These species have the ability to rapidly evolve through horizontal gene transfer (e.g., mobile genetic elements) (1). This includes the ability to develop resistance to multiple antibiotics, which complicates the treatment of infections and increases, potentially mortality and morbidity in patients. Of particular concern is the emergence of carbapenem-resistant Enterobacterales (CR-E), which poses a major concern in clinical as well as community settings all across the globe (2). Carbapenems referred to as last-resort antibiotics, possess a broad spectrum of activity against most clinically-relevant Gram-negative bacteria (3, 4). Consequently, it is crucial to continuously monitor and assess the spread of carbapenem resistance, especially in low- and middle-income countries (LMICs) that face established challenges in antimicrobial stewardship and public health systems.

Available observations suggest that antimicrobial resistance (AMR) has been precipitously increasing in Lebanon, a country with a plethora of issues resulting from an unprecedented economic collapse (5–7). The latter has amplified critical issues such as access to medical care, sanitation, and nutritious and safe food, as well as promoted lax medical practices, including self-medication and the reliance on widely and easily available antibiotics as cheaper alternatives across the country. This is important because excessive and inappropriate use of antimicrobials in human and veterinary medicine and agriculture has been well-documented in Lebanon, even before the economic collapse (8–10). Taken together, these challenging conditions have been predicted to enhance the emergence of AMR and the cycle of complicated infections, especially in the most vulnerable populations in Lebanon (5–7, 10, 11). Nevertheless, studies on AMR in Lebanon are generally scant and, when available, can be limited, in scope or (e.g., low sample number) and/or descriptive (e.g., phenotypic AMR evaluation and absence of in-depth genomic analysis). Despite this, available studies have reported rates of extended-spectrum cephalosporin-resistant Enterobacterales (ESCR-E; ~50% of tested isolates) and CR-E (~3%) among clinical isolates in Lebanon between 2015 and 2019 (12, 13). The enzymatic nature of the carbapenem-resistance has also been evidenced, by the detection of several carbapenemase genes, including *bla*_OXA-48_, *bla*_NDM-5_, and *bla*_NDM-19_, in *Escherichia coli*, *bla*_OXA-48_, *bla*_OXA-181_, and *bla*_NDM-5_ in *Klebsiella pneumoniae*, *bla*_OXA-48_, *bla*_VIM-1_, *bla*_VIM-4_, and *bla*_NDM-1_ in *Enterobacter cloacae*, and *bla*_OXA-48_ in *Citrobacter freundii* isolated in Lebanese hospitals (12). In addition, high rates of multidrug-resistant (MDR) Enterobacterales (60.7%) and the dissemination of extended-spectrum ß-lactamase (ESBL) producing-, carbapenemase-producing (CP)-, and colistin-resistant *E. coli* isolates among healthy people in the Lebanese community (14), animals (15), and the environment (16) has been documented. Hence, in-depth studies to evaluate the emergence and spread of AMR in Lebanon, focusing on the determinants that contribute to the dissemination of ESBL-E and/or CP-E is now mandatory. Specifically, details of the sequence types of these bacteria, their resistome, and the plasmids carrying these genes may be crucial to identify the transmission routes and propose intervention strategies to limit their spread. To fill these gaps, we evaluated the occurrence of ESBL- and/or CP-E in three different hospitals in Lebanon and determined the underlying mechanisms of resistance, the population structure of the isolates, and the associated plasmid types using whole genome sequencing analysis (WGS), a powerful technique for investigating AMR, but still not commonly available in LMICs, such as Lebanon, due to its relative expensiveness and requirement of specialized equipment and skills (17).

This study focused only on hospital isolates, as (1) anecdotal evidence suggests that hospitals play an important role in the transmission of ESBL-E- and CP-E, and (2) hospitalized patients are more susceptible to infections/colonization with these species.

## 2 Materials and methods

### 2.1 Ethical approval

This study was approved by the Azm Center/Lebanese University ethical committee and the Lebanese Ministry of Public Health (CE-EDST-1-2020). All the specimens were analyzed anonymously, without any patient identifiers, and the patients were not physically involved in this study.

### 2.2 Isolation and identification of bacteria from clinical samples

A total of 188 clinical Enterobacterales isolates being either resistant to Expanded Spectrum Cephalosporin (ESCR) or Carbapenem Resistant were collected between 2019 and 2020 by the bacteriology laboratories of three hospitals, including El Youssef Hospital Center (50 isolates), the Nini Hospital (137 isolates), and the Tripoli Governmental Hospital (1 isolate), which are located in the Akkar and North governorates of Lebanon, respectively. These isolates were collected based on their susceptibility profile established in the hospitals as part of routine clinical testing. These clinical isolates were recovered from different sample types, including urine, pus, wound, rectal, axillary, pleural fluid, gastric fluid, and bronchial fluid. They were identified at the hospitals using the matrix-assisted laser desorption/ionization time-of-flight (MALDI-TOF) with VITEK MS protocol (bioMérieux, Version 3.0, Marcy L’Etoile, France). The isolates were subsequently stored at the Lebanese University bacterial bank (CMUL).

### 2.3 Antimicrobial susceptibility testing

The isolates were screened for ESBL and CR phenotypes using the Kirby-Bauer disk diffusion assay (including Ticarcillin, Ticarcillin/clavulanic acid, cefoxitin, ceftazidime, cefepime, temocillin, and imipenem). As for the CR-E isolates, a total of 15 β-lactams (amoxicillin, amoxicillin/clavulanic acid, ticarcillin, ticarcillin/clavulanic acid, piperacillin, piperacillin/tazobactam, cefoxitin, cefotaxime, cefepime, ceftazidime, aztreonam, ertapenem, imipenem, meropenem, and temocillin) antibiotics of human interest were tested. The minimum inhibitory concentrations (MICs) were also determined by E-test (bioMérieux) for ertapenem, imipenem, and meropenem, while temocillin and colistin MICs were assessed using the broth microdilution method.

Additionally, 13 CP-*E. coli* isolates were selected according to their resistance phenotype, genotype, and MLST type for further determination of MIC values using broth microdilution (BMD) test (Sensititre, ThermoFisher, Grenoble, France) for a complementary list of novel beta-lactam and non-beta-lactam antibiotics of clinical and veterinary interest (i.e., ceftazidime/avibactam, ceftiofur, ceftaroline, ceftobiprole, aztreonam-avibactam, mecillinam, imipenem/relebactam, meropenem/vaborbactam, cefiderocol, eravacyclin, apramycin, gentamicin, neomycin, streptomycin, sulfonamides, and nitrofurantoin). Susceptibility patterns were interpreted according to the European Committee on Antimicrobial Susceptibility Testing (EUCAST)^1^ guidelines when available (18). For aztreonam/avibactam, interpretation was done using aztreonam breakpoints alone (18). For ceftiofur, apramycin, neomycin and streptomycin veterinary breakpoints were used.^2^

### 2.4 Evaluation of the ESCR- and CR-E isolates using enzymatic assays

The β LACTA^™^ test (Bio-Rad, Marne-la-Coquette, France) was used to further evaluate the ESCR-E isolates. Briefly, this test is based on the hydrolysis of a chromogenic cephalosporin that turns red upon hydrolysis. Notably, the chromogenic cephalosporin is not hydrolyzed by acquired penicillinases (e.g., SHV-1, TEM-1) but by ESBL, carbapenemase, and acquired AmpC (19). Furthermore, the NG-Test^®^ CTX-M MULTI (NG Biotech, Guipry, France) immunochromatographic assay (ICA) was performed on the β LACTA^™^ positive isolates to infer the presence of CTX-M-type ESBLs (20). The CR-E isolates were also evaluated using the Carba NP hydrolysis test, which detects carbapenemase activity based on *in vitro* hydrolysis of imipenem (21). The NG-Test^®^ CARBA-5 ICA (NG Biotech) was used to detect members of the five main families of carbapenemases (i.e., KPC-, NDM-, VIM-, IMP-, and OXA-48-like enzymes) produced by the CR-E isolates as described in the manufacturers’ instructions (22).

### 2.5 Molecular characterization of the ESCR- and CR-E isolates

The ESCR-E isolates (positive using the β-LACTA^™^ and NG-Test^®^ CTX-M MULTI tests) were screened by PCR and subsequent Sanger sequencing to identify the *bla*_CTX-M_ allele as described previously (23, 24). For the ESCR-E isolates (positive using the β-LACTA^™^ but negative with the NG-Test^®^ CTX-M MULTI) and the CR-E isolates, the total DNA was extracted using the PureLink^™^ Genomic DNA Mini-Kit (ThermoFisher Scientific, Waltham, MA, United States) following the manufacturer’s instructions and stored at -20°C. Genomic DNA was used for library preparation using the NEBNext Ultra II FS DNA Library Prep Kit for Illumina (NEB, France) according to the manufacturer’s instructions. Whole genome sequencing was performed on an Illumina NextSeq 500 instrument (Illumina). After sequencing, raw data were assembled *de novo* using the CLC genomics 10.2 program (Qiagen, Les Ulis, France), and the genomes were analyzed online using software available at the Center for Genomic Epidemiology-CGE.^3^ The latter included multilocus sequence typing (MLST) with the CGE MLST 2.0 software to determine sequence types (ST), and acquired resistance gene determinations using ResFinder 4.1 (25–27). Similarly, plasmid replicon types and virulence genes were identified using PlasmidFinder 2.0 (26, 28) and VirulenceFinder 2.0., respectively (26, 29, 30). Reference plasmids sequence were retrieved from the NCBI database, using a local BLAST algorithm. Reads and/or Contigs carrying carbapenemase genes were mapped to reference plasmids, using the CLC genomics 10.2 program (Qiagen).

### 2.6 Plasmids from CR-E isolates

Plasmids were extracted from the carbapenem-resistant isolates by the Kieser method as previously described (31). Transfer of plasmid-borne resistance markers was assessed by electroporation of the plasmids into electro-competent *E. coli* TOP10 (Invitrogen, Saint-Aubin, France). Transformants were selected on Trypticase soy agar (TSA) supplemented with ticarcillin (100 μg/mL). Transformants were PCR screened for the carbapenemase genes likely transferred to the recipient *E. coli*. Plasmids were visualized using electrophoresis on 0.7% agarose. *E. coli* NCTC 50192, which harbors four plasmids (7, 48, 66, and 154 kb) was used as size markers during electrophoresis (31).

### 2.7 Typing of the CR-E isolates using infrared spectrometry

The CR-E isolates were typed using the Bruker IR Biotyper spectrometer (IRBT, Bruker, Hamburg, Germany). An amount of 1 μL of bacterial colonies selected from the confluent part of the culture was re-suspended in 50 μL of 70% ethanol solution in an IR Biotyper suspension vial. After vortexing, 50 μL of deionized water was added, and the solution was mixed by pipetting. The bacterial suspensions (15 μL) were spotted in three technical replicates onto the 96-spot silicon IR Biotyper target and let dry for 15–20 min at 35°C ± 2°C. In each run, prior to sample spectra acquisition, quality control was performed with the Infrared Test Standards (IRTS 1 and 2) of the IR Biotyper kit. IRTS 1 and IRTS 2 were re-suspended in 90 μL deionized water and 90 μL of absolute ethanol was added and mixed. Subsequently, 12 μL of the suspension was spotted onto the IR Biotyper target and let dry as previously described. The relationships between the isolates were analyzed using the Bruker IR Biotyper Software (version 2.1.0.195, Bruker) (32). An online tool^4^ was used to assess the quantitative data of discriminatory power and concordance of the typing methods. Simpson’s index of diversity (SID) was used to evaluate the discriminatory power of the typing method, calculating the probability that two unrelated isolates from the test strain set will be clustered into different typing groups. Adjusted Rand index (ARI) with 95% confidence intervals was used to evaluate the concordance of IRBT typing results (33).

## 3 Results

### 3.1 Bacterial isolates

Based on the susceptibility profile (Resistance to ESC and/or carbapenems) derived from the microbiology laboratories of the three participating hospitals, 188 isolates, *E. coli* (*n* = 151; 80.3%), *K. pneumoniae* (*n* = 20; 10.6%), *Enterobacter* spp. (*n* = 7; 3.7%), *Proteus mirabilis* (*n* = 6; 3.2%), *Salmonella* spp. (*n* = 3; 1.6%), and *Serratia marcescens* (*n* = 1; 0.5%) were collected. Using the Kirby-Bauer disk diffusion assay, only 90 isolates could be confirmed and were included in this study. This collection comprised *E. coli* (*n* = 70; 77.8%), *K. pneumoniae* (*n* = 13; 14.4%), *Enterobacter* spp. (*n* = 6; 6.7%), and *Proteus mirabilis* (*n* = 1; 1.1%). Routine antimicrobial susceptibility testing revealed that out of these 90 isolates, 57 were only ESBL producers (e.g., fully susceptible to carbapenems), while the remaining 33 (i.e., 26 *E. coli*, five *K. pneumoniae*, one *E. cloacae*, and one *Enterobacter hormaechei*) were resistant to at least one carbapenem, of which 20 were also ESBL-producers, as revealed by synergy images between ESCs and clavulanic acid containing disks.

### 3.2 Susceptibility testing and ESBL gene characterization among the ESBL-producers

The 57 ESBL-producing isolates (Table S1) were composed of *E. coli* (*n* = 44; 77.2%), *K. pneumoniae* (*n* = 8; 14%), *Enterobacter* spp. (*n* = 4; 7%), and *Proteus mirabilis* (*n* = 1; 1.8%). These isolates were MDR and remained consistently susceptible only to carbapenems.

The NG-Test^®^ CTX-M MULTI showed that all but two isolates were CTX-M positive (Table S1). PCR amplification of the entire *bla*_CTX-M_-gene and subsequent sequencing revealed that *bla*_CTX-M-15_ was predominant (91.2%, 52/55), followed by *bla*_CTX-M-55_ (3.5%, 2/55) and *bla*_CTX-M-3_ gene (1.8%, 1/55). The whole genome sequencing analysis showed that the two non-CTX-M producing ESBL isolates: one *E. coli* isolate harboring a *bla*_SHV-12_ ESBL gene and one *K. pneumoniae* isolate harboring a chromosomally encoded *bla*_SHV-187_ gene.

### 3.3 Carbapenemase detection and susceptibility testing of CR-E isolates

The Carba NP hydrolysis test and the NG-Test^®^ CARBA-5, are displayed in Table 1 and Table S2. Among the 33 CR-E isolates, 23 were positive using the Carba NP test, while the remaining isolates were repeatedly negative. Using the NG-Test^®^ CARBA-5 ICA, all 33 isolates were CPs: 17 isolates (51.5%) were positive for NDM and 16 (48.5%) for OXA-48-like enzymes. Out of the 26 *E. coli* isolates different AMR phenotypes were observed (Figure S1). NDM (*n* = 14) and OXA-48-like (*n* = 12) were the only carbapenemases detected in these isolates. Three *K. pneumoniae* isolates were positive for OXA-48-like, and two for NDM. The two *E. cloacae* complex isolates produced either an OXA-48-like or an NDM.

**Table 1:** Antibiotic susceptibility patterns using Kirby-Bauer disk diffusion of carbapenemase-producing Enterobacterales (expect colistin).

Isolate code	Sample	Hospital	CarbaNP	NG-Test Carba5	β LACTA	NG-Test CTX-M MULTI^!^	FOX	CAZ	CTX	ATM	FEP	IMP	MEM	FDC	MEC	FSF	TGC	CIP	LVX	CHL	SXT	FUR	TMN	AMK	GMN	CST
***Escherichia coli***																										
O84C6	Urine	YHC	+	OXA-like	+	−	R	R	R	R	R	S	S	S	S	S	S	S	S	R	R	S	S	S	S	2
O84C10	Gastric fluid	YHC	−	OXA-like	+	+	S	R	R	R	R	S	S	S	R	S	S	S	S	S	R	S	S	S	S	4
O84D2	Urine	YHC	+	OXA-like	−	ND	S	S	S	S	S	S	S	S	S	S	S	S	S	S	R	S	S	S	S	1
O84D6	Urine	YHC	−	OXA-like	+	+	S	S	S	S	S	S	S	S	R	S	S	S	S	S	R	S	S	S	S	1
O84E5	Urine	YHC	−	OXA-like	−	ND	R	R	R	S	S	S	S	S	S	S	S	S	S	S	R	S	S	S	S	1
O84E7	Urine	YHC	+	NDM	−	ND	R	R	R	R	R	R	R	R	R	S	S	R	R	R	R	S	R	R	R	2
O84E8	Urine	YHC	−	OXA-like	−	ND	R	R	R	S	S	S	S	S	S	S	S	S	S	S	S	S	S	S	S	1
O84E9	Urine	NH	+	NDM	+	+	R	R	R	R	R	R	R	S	R	R	S	R	R	R	R	S	R	R	R	2
O84F2	Urine	YHC	−	OXA-like	+	+	S	R	R	R	R	S	S	S	S	S	S	S	S	S	S	S	S	S	S	1
O84F5	Urine	YHC	+	NDM	+	−	R	R	R	R	R	R	R	R	R	S	S	R	R	R	R	S	S	S	S	0.5
O86D2	Urine	YHC	+	NDM	+	+	R	R	R	R	R	R	R	S	R	S	S	R	R	R	R	S	R	S	R	1
O84F9	Urine	YHC	+	NDM	−	ND	R	R	R	R	R	R	R	R	R	S	S	R	R	R	R	S	S	S	S	1
O84G8	Urine	YHC	−	OXA-like	−	ND	R	R	R	S	S	S	S	S	S	S	S	S	S	S	R	S	S	S	S	1
O85A1	Urine	YHC	−	OXA-like	+	+	S	R	R	R	R	S	S	S	S	S	S	R	R	S	R	S	R	S	R	1
O85A3	Urine	NH	−	OXA-like	−	ND	S	S	S	S	S	S	S	S	S	S	S	S	S	S	S	S	S	S	S	1
O85C3	Urine	YHC	+	NDM	+	+	R	R	R	R	R	R	R	R	R	S	S	R	R	R	R	R	R	R	R	2
O85C6	Urine	YHC	+	NDM	+	+	R	R	R	R	R	R	R	R	S	S	S	R	R	S	R	S	S	S	S	2
O85C10	Urine	YHC	+	NDM	+	+	R	R	R	R	R	R	R	R	S	S	S	R	R	R	R	R	R	S	S	0.5
O85D6	Pleural fluid	NH	+	NDM	−	ND	R	R	R	S	R	R	R	R	R	S	S	R	R	S	R	S	S	S	S	1
O85D8	Urine	YHC	−	OXA-like	−	ND	S	R	S	S	S	S	S	S	R	S	S	S	S	S	S	S	S	S	S	0.5
O85E2	Axillary	NH	+	NDM	−	ND	R	R	R	S	R	R	R	R	R	S	S	R	R	S	R	S	S	S	S	2
O85F3	Pus	NH	+	NDM	−	ND	R	R	R	S	R	R	R	R	R	S	S	R	R	S	R	S	S	S	S	1
O85G1	Wound	NH	+	NDM	−	ND	R	R	R	S	R	R	R	S	R	S	S	R	R	S	R	S	S	S	S	2
O86A2	Urine	NH	−	OXA-like	−	ND	S	S	S	S	S	S	S	S	S	S	S	S	S	S	S	S	S	S	S	2
O85C4	Urine	NH	+	NDM	+	+	R	R	R	R	R	R	R	R	S	S	S	R	R	S	R	S	R	S	S	1
O86A6	Rectal	NH	+	NDM	+	+	R	R	R	R	R	R	R	R	R	S	S	R	R	S	R	S	R	S	S	1
***Klebsiella pneumoniae***																										
O84C9	Blood culture	TGH	+	NDM	+	+	R	R	R	S	R	S	R	R	R	S	S	S	S	S	R	S	R	R	R	1
O84D5	Pus	YHC	+	OXA-like	+	−	R	R	R	R	R	S	R	S	R	R	S	S	S	R	S	R	R	R	R	1
O84E1	Gastric fluid	YHC	+	OXA-like	+	+	S	R	R	R	R	S	S	S	S	S	S	S	S	S	S	R	R	R	R	1
O85D10	Urine	NH	+	NDM	+	+	R	R	R	R	R	S	S	S	S	R	S	R	S	R	R	S	R	R	R	2
O85E3	Axillary	NH	+	OXA-like	+	+	R	R	R	R	R	S	R	S	S	R	S	S	S	R	S	S	S	S	S	2
***Enterobacter* spp.**																										
O84D8^!!^	Urine	NH	+	NDM	+	−	R	R	R	S	R	S	R	R	R	S	S	R	R	R	R	R	R	R	R	1
O84F3^!!^	Urine	YHC	+	OXA-like	+	−	R	R	R	R	R	S	R	S	S	R	S	S	S	S	S	S	S	S	S	1

*Susceptibility results were interpreted according to the EUCAST guidelines All isolates are resistant to amoxicillin, amoxicillin/Clavulanate, Ticarcillin, Ticarcillin/Clavulanate, piperacillin, piperacillin/tazobactam, temocillin, and ertapenem; (18). Gray: Resistant; No color: Susceptible; Abbreviations: FOX, cefoxitin; CAZ, ceftazidime; CTX, cefotaxime; ATM, aztreonam; FEP, Cefepim; IMP, imipenem; MEM, meropenem; FDC, Cefiderocol; MEC, Mecillinam; FSF, Fosfomycin; TGC, Tigecycline; CIP, ciprofloxacin; LVX, levofloxacin; CHL, chloramphenicol; SXT, trimethoprim-sulfamethoxazole; FUR, nitrofurantoin; TMN, tobramycin; AMK, Amikacin; GMN, gentamicin; CST, colistin; YHC, El Youssef Hospital Center; TGH, Tripoli Governmental Hospital; NH, Nini Hospital. Colistin MIC was assessed using the broth microdilution method according to the EUCAST guidelines (18). ^!^The NG-Test CTX-M MULTI was only performed on positive β LACTA isolates. ^!!^O84D8, Enterobacter hormaechei; O84F3, Enterobacter cloacae (as identified by MALDI-TOF MS).

Using disk diffusion antibiograms, all CR-E isolates were found to be resistant to nearly all antibiotics tested routinely in Lebanon, including ertapenem (*n* = 33; 100%). However, 31% (8/26) of these *E. coli* isolates were found susceptible to ertapenem (i.e., MIC ≤0.5 mg/L) using the broth microdilution method; of them, six isolates were negative with the Carba NP test, while all were positive by ICA for OXA-48. While NDM- and some OXA-48-like- producing *E. coli* displayed high MIC levels for temocillin, surprisingly most isolates (75%, 9/12) carrying *bla*_OXA-48-like_ had relatively low MICs (≤ 64 μg/mL) for this antibiotic (as compared to 14.3%; 2/14) of the NDM-producing *E. coli* isolates.

Colistin MIC results revealed that almost all CR-E isolates (97%, 32/33) remained consistently susceptible to this antibiotic (MIC ≤2 mg/L). Furthermore, the activity of different last-resort beta-lactam and non-beta-lactam antibiotics was assessed against a subpopulation of CR-*E. coli* isolates (6 *bla*_NDM_ and 7 *bla*_OXA-48-like_-producers). All the tested isolates were resistant to ceftaroline and ceftobiprole but susceptible to eravacyclin, nitrofurantoin, apramycin, and tigecycline. Resistance to cefiderocol, ceftazidime/avibactam, imipenem/relebactam, and meropenem/vaborbactam was observed among the *bla*_NDM_-producing isolates (Table 2). Additionally, increased MICs to aztreonam/avibactam (4, 8, and 16 mg/L) were noticed among *bla*_NDM_-producing *E. coli* isolates.

**Table 2:** Antibiotic susceptibility patterns of selected carbapenemase-producing *Escherichia coli* isolates for antibiotics considered last resort molecules using broth microdilution test (Sensititre), and interpreted according to the EUCAST guidelines (18).

Isolate code	MLST	Carbapenemase gene	Amino acids insertion in PBP3	CAZ/AVI	CEF	CFT	BPR	FDC	ATM/AVI^1^	IMP/REL	MER/VAB	ERV	APR	NEO	STR	SA
O84C6	ST940	OXA-181	–	S	R	R	R	S	I	S	S	S	S	S	R	S
O84C10	ST46	OXA-244	–	S	R	R	R	S	S	S	S	S	S	S	R	R
O84D2	ST69	OXA-48	–	S	S	R	R	S	S	S	S	S	S	S	R	R
O84D6	ST10	OXA-244	–	S	R	R	R	S	S	S	S	S	S	S	R	R
O84E7	ST405	NDM-5	YRIK	R	R	R	R	R	I	R	R	S	S	S	R	R
O84E9	ST405	NDM-5	YRIK	R	R	R	R	R	I	R	R	S	S	R	R	R
O84F2	ST38	OXA-244	–	S	R	R	R	S	S	S	S	S	S	S	S	S
O86D2	ST90	NDM-5	–	R	R	R	R	S	S	R	R	S	S	R	R	R
O84F9	ST631	NDM-5	YRIN	R	R	R	R	S	I	R	R	S	S	S	R	R
O85A1	ST8881	OXA-244	–	S	R	R	R	S	S	S	S	S	S	S	R	R
O85C6	ST167	NDM-5	YRIN	R	R	R	R	R	I	R	S	S	S	S	R	R
O85E2	ST648	NDM-5	YRIK	R	R	R	R	R	I	R	R	S	S	S	R	R
O86A2	ST69	OXA-244	–	S	S	R	R	S	S	S	S	S	S	S	S	S

The isolate selection was done according to their resistance phenotype, genotype and MLST type. *Dark gray: Resistant; Light gray: Intermediate non-susceptible; No color: Susceptible; Abbreviations: CAZ/AVI, ceftazidime/avibactam; CEF, Ceftiofure; CFT: ceftarolin; BPR, ceftobiprole; FDC: Cefiderocol; ATM/AVI, aztreonam/avibactam; IMP/REL, imipenem/relebactam; MEM/VAB, meropenem/vaborbactam, ERV, eravacyclin; APR, apramycin; NEO, neomycin; STR, streptomycin; SA, sulfonamides.^1^ As no breakpoints are yet available, those of ATM were used.

The whole genome sequencing of the 33 CR-E isolates was performed. After read assembly, the contigs were submitted to ResFinder 4.0. In total, 47 different resistance genes were identified (Table S3), which code for resistance determinants to clinically-important classes of antibiotics, including β-lactams, aminoglycosides, tetracycline, quinolones, trimethoprim-sulfamethoxazole, fosfomycin, and sulfonamides. Among *E. coli* isolates, the most frequently identified carbapenemase was NDM-5 (53.8%; 14/26), followed by OXA-244 (38.5%; 10/26), OXA-181 (3.8%; 1/26), and OXA-48 (3.8%, 1/26). OXA-48 has also been identified in three *K. pneumoniae* and one *E. cloacae* isolates, while NDM-1 (*n* = 3, 9.1%) was detected in two *K. pneumoniae* and one *E. hormaechei* isolates (Table 2). Notably, two plasmid-encoded cephalosporinases, *bla*_CMY-145_ and *bla*_DHA-1_ genes that confer resistance to ESCs were detected in three *E. coli* isolates. ESBL genes such as *bla*_CTX-M-15_ (*n* = 15), *bla*_CTX-M-27_ (*n* = 1), *bla*_TEM-35_ (*n* = 1)_,_ *bla*_SHV-33_ (*n* = 1), *bla*_SHV-12_ (*n* = 2)_,_ *bla*_SHV-26_ (*n* = 1), were also identified. Specifically, the *bla*_CTX-M-15_ gene was the most frequently detected in *E. coli* (*n* = 11; including one isolate co-harboring two CTX-M alleles: *bla*_CTX-M-15_ and *bla*_CTX-M-27_) and *K. pneumoniae* (*n* = 4) isolates. 16S RNA methylase genes were detected among 6 isolates including 5 *bla*_NDM-5-_producing *E. coli* and one *bla*_NDM-1_-producing *E. hormaechei* isolate.

MLST analysis using the whole genome sequence showed that the CR-*E. coli* belonged to 11 different STs; including ST69 (*n* = 6 isolates), followed by ST648 (*n* = 4), ST167 (*n* = 3), ST361 (*n* = 3), ST405 (*n* = 3), ST10 (*n* = 2), ST90 (*n* = 1), ST940 (*n* = 1), ST38 (*n* = 1), ST46 (*n* = 1), and ST8881 (*n* = 1). Additionally, the CR-*K. pneumoniae* isolates belonged to four different STs, namely ST35, ST37, ST45, and ST1770. *E. cloacae* and *E. hormaechei* belonged to ST1006 and ST182, respectively (Table S3).

Furthermore, alignment of the *ftsI* gene sequences with that of a wild-type gene (*E. coli* NCTC 9022, accession number LR134237) revealed a four amino-acid insertion in PBP3 after residue 333 in 13 of 14 NDM-5-producing *E. coli* isolates. Two types of insertions were detected: YRIN (*n* = 7) and YRIK (*n* = 6). YRIN insertion was found among isolates with ST167 and ST361, and YRIK insertion was present in isolates with ST648 and ST405. ST90 isolate contained neither YRIN nor YRIK insertions. These 4 AA insertions could be correlated with increased MICs to aztreonam/avibactam (4, 8, and 16 mg/L) and cefiderocol among *bla*_NDM-5_ producing *E. coli* isolates, as compared to similar isolates lacking a 4 AA insertion (0.06 mg/mL).

### 3.4 Virulence determinants and plasmids in the CR-*Escherichia coli* isolates

Virulence factors (VFs) in the CR-*E. coli* isolates were identified using the CGE VirulenceFinder 2.0. TraT protein, previously shown to mediate resistance to bacterial killing by serum, was detected in 14 *E. coli* isolates, including a *bla*_OXA-48_-positive isolate belonging to ST69. Genes encoding adhesins (*ipfA, fimH, afaA, afaC, afaD, afaE*) were found in five *E. coli* that were positive for *bla*_OXA-244_ (Table 3). Additionally, *iss* encoding an outer membrane lipoprotein that enhances serum resistance, was detected only in one *bla*_OXA-244_-positive *E. coli* isolate. The capsular genes, *kpsE* and *kpsM*, were both detected in eight isolates, of which three were *bla*_NDM-5_-positive (ST648), and 5 were *bla*_OXA-244_-positive (ST69). Most VFs were detected in two isolates belonging to ST69.

**Table 3:** Virulence genes of the 26 carbapenemase-producing *E. coli* clinical isolates.

				Adhesion						Siderophore		Serum resistance		Invasive	Capsular			Other genes															
Isolate code	Sample	Carbapenemase gene	MLST type	*ipfA*	*fimH*	*afaA*	*afaC*	*afaD*	*AfaE*	*iucC*	*iutA*	*traT*	*iss*	*eilA*	*capU*	*kpsE*	*kpsMII*	*fyuA*	*terC*	*chuA*	*irp2*	*ompT*	*traJ*	*hra*	*iha*	*sitA*	*papC*	*yfcv*	*csgA*	*hlyE*	*cia*	*cib*	*sat*
O84D6**	Urine	*bla*_OXA-244_	ST10																														
O85D8**	Urine	*bla*_OXA-244_	ST10																														
O84F2**	Urine	*bla*_OXA-244_	ST38																														
O84C10**	Gastric Fluid	*bla*_OXA-244_	ST46																														
O84D2**	Urine	*bla*_OXA-48_	ST69																														
O84E5**	Urine	*bla*_OXA-244_	ST69																														
O84E8**	Urine	*bla*_OXA-244_	ST69																														
O84G8**	Urine	*bla*_OXA-244_	ST69																														
O85A3*	Urine	*bla*_OXA-244_	ST69																														
O86A2*	Urine	*bla*_OXA-244_	ST69																														
O86D2**	Urine	*bla*_NDM-5_	ST90																														
O85C6**	Urine	*bla*_NDM-5_	ST167																														
O85C4*	Urine	*bla*_NDM-5_	ST167																														
O85C10**	Urine	*bla*_NDM-5_	ST167																														
O84F5**	Urine	*bla*_NDM-5_	ST361																														
O84F9**	Urine	*bla*_NDM-5_	ST361																														
O86A6*	Rectal	*bla*_NDM-5_	ST361																														
O84E7*	Urine	*bla*_NDM-5_	ST405																														
O84E9*	Urine	*bla*_NDM-5_	ST405																														
O85C3**	Urine	*bla*_NDM-5_	ST405																														
O85E2*	Axillary	*bla*_NDM-5_	ST648																														
O85F3*	Pus	*bla*_NDM-5_	ST648																														
O85G1*	Wound	*bla*_NDM-5_	ST648																														
O85D6*	Pleural Fluid	*bla*_NDM-5_	ST648																														
O85A1**	Urine	*bla*_OXA-244_	ST8881																														
O84C6**	Urine	*bla*_OXA-181_	ST940																														

Virulence genes were detected using the VirulenceFinder 2.0 online tool available at the Center for Genomic Epideiology-CGE (http://www.genomicepidemiology.org/) (26, 29, 30). Virulence genes were detected using 100% identity as cut-off. *Nini Hospital; **El-Youssef Hospital Center; ***Tripoli Governmental Hospital; Different colors represent different sequence types (STs).

Using PlasmidFinder 2.1, 9 plasmid replicon types were identified. Specifically, the following plasmid types were detected in the *E. coli* isolates; Col (*n* = 9 isolates), IncFII (*n* = 18), IncX3/X4 (*n* = 4), IncI1-I (*n* = 3), and IncFIA (*n* = 11). Additionally, in the *K. pneumoniae* isolates, IncL (*n* = 3), IncM2 (*n* = 1), and IncFIB (*n* = 4) were detected. The *E. cloacae* and *E. hormaechei* isolates carried IncFIB/II and IncL/X3, respectively (Table 4). Transformants were obtained after Kieser plasmid extraction and electro-transformation for 25/33 isolates. For 8 *bla*_OXA-244_- producing isolates, even with repeated attempts no plasmids were observed on Kieser gel, and no transformants were obtained suggesting a chromosomal location. For *K. pneumoniae* and *E. hormaechi*, the *bla*_OXA-48_ gene was carried on an IncL plasmid.

**Table 4:** Carbapenemase gene location was determined by plasmid extraction using Kieser technique and by applying a BLASTN algorithm for carbapenemase-genes-carrying contigs and then these contigs were mapped to reference plasmids using CLC genomics.

Isolate code	MLST type	Carbapenemase gene	Location (chromosomal or plasmid-borne)	IncX3	IncX4	IncFIA	IncFIB	IncFII	Col	Incl1-I	IncL	IncM
*Escherichia coli*												
O84D6**	ST10	*bla*_OXA-244_	P									
O85D8**	ST10	*bla*_OXA-244_	C									
O84F2**	ST38	*bla*_OXA-244_	C									
O84C10**	ST46	*bla*_OXA-244_	C									
O84D2**	ST69	*bla*_OXA-48_	P									
O84E5**	ST69	*bla*_OXA-244_	C									
O84E8**	ST69	*bla*_OXA-244_	C									
O84G8**	ST69	*bla*_OXA-244_	C									
O85A3*	ST69	*bla*_OXA-244_	C									
O86A2*	ST69	*bla*_OXA-244_	C									
O86D2**	ST90	*bla*_NDM-5_	P									
O85C6**	ST167	*bla*_NDM-5_	P									
O85C4*	ST167	*bla*_NDM-5_	P									
O85C10**	ST167	*bla*_NDM-5_	P									
O84F5**	ST361	*bla*_NDM-5_	P									
O84F9**	ST361	*bla*_NDM-5_	P									
O86A6*	ST361	*bla*_NDM-5_	P									
O84E7*	ST405	*bla*_NDM-5_	P									
O84E9*	ST405	*bla*_NDM-5_	P									
O85C3**	ST405	*bla*_NDM-5_	P									
O85E2*	ST648	*bla*_NDM-5_	P									
O85F3*	ST648	*bla*_NDM-5_	P									
O85G1*	ST648	*bla*_NDM-5_	P									
O85D6*	ST648	*bla*_NDM-5_	P									
O85A1**	ST8881	*bla*_OXA-244_	C									
O84C6**	ST940	*bla*_OXA-181_	P									
*Klebsiella pneumoniae*												
O84E1**	ST35	*bla*_OXA-48_	P									
O84D5**	ST35	*bla*_OXA-48_	P									
O85E3*	ST37	*bla*_OXA-48_	P									
O84C9***	ST45	*bla*_NDM-1_	P									
O85D10*	ST1770	*bla*_NDM-1_	P									
*Enterobacter* spp.												
O84D8*	ST182	*bla*_NDM-5_	P									
O84F3**	ST1006	*bla*_OXA-48_	P									

Plasmid incompatibility groups present in the 33 clinical carbapenemase-producing Enterobacterales isolates, were identified using PlasmidFinder 2.1 online tool available at the Center for Genomic Epidemiology -CGE (http://www.genomicepidemiology.org/) using 99% identity cut-off (26, 28–30).*Nini Hospital; **El-Youssef Hospital Center; ***Tripoli Governmental Hospital; Different colors represent different sequence types (STs).

### 3.5 Genetic relatedness as revealed by IR Biotyper in comparison to WGS

Sixteen CR-*E. coli* and the five CR-*K. pneumoniae* isolates positive with the Carba NP test were assessed with the IR Biotyper for strain typing and the results were compared with those obtained by WGS analysis (Figures S2, S3). Overall IRBT results corroborated WGS for the typing of the five CR *K. pneumoniae,* which were classified into four IR types (Figure S2). However, IRBT differentiated 16 *E. coli* isolates that belonged to 7 STs into 11 IR types, with 8 IR types comprising only a single isolate (Table 5). The Simpson Index of Diversity (SID) was used to determine the discriminatory power of the typing methods. WGS had the highest discriminatory power (0.967) followed by IRBT (0.933), while the lowest SID was for MLST (0.875). Main discrepancies were observed between IRBT and WGS with *E. coli* ST-648 isolates (O85D6, O85E2, O85G1, and O85F3), which clustered into IR type 7 (Figure S3), but differed from each other by 53, 400, and 1,360 SNIPs.

**Table 5:** Comparison of different typing methods for carbapenemase-producing *Escherichia coli* clinical isolates.

Method	No. of types	Simpson’s ID (95% CI)
MLST^a^	7	0.875 (0.813–0.937)
IRBT^b^	11	0.933 (0.857–1.000)
WGS^c^	12	0.967 (0.935–0.998)

Simpson’s index of diversity used to determine the discriminatory power and concordance of the three typing methods was determined using the online tool 690 (www.comparingpartitions.info). ^a^MLST: Multi-locus sequence typing; ^b^IRBT: IR Biotyper; ^c^WGS: Whole-genome sequencing.

## 4 Discussion

The increase in ESCR- and CR-E isolates has been observed in hospitals worldwide. In Lebanon, two nationwide hospital-based retrospective studies documented a high prevalence of MDR pathogens, including ESBL-producing Enterobacterales (34% in 2016) (5). Recently, a community-based study revealed that approximately half of the population carries ESCR-*E. coli* (14), with an important dynamic of acquisition and loss of MDR strains and limited plasmid spread. The occurrence of Enterobacterales with decreased susceptibility to carbapenems, raised from 0.4% in 2008–2010 to 1.6% in 2012 and 3.3% in 2019 in hospitalized patients (12, 35). Despite the concerning increase and reports, there is a lack of molecular data on the epidemiology of ESBL- and CR-E in Lebanon.

Our results highlighted the predominance of the *bla*_CTX-M-15_ gene (52/57, 91.2%) among ESBL-producing isolates regardless of the co-existence of other ß-lactam resistance determinants, as observed globally (36). CTX-M-15-producing Enterobacterales clones have been widely reported in Lebanon among human, animal, and environmental sources (14, 15, 37–39). The finding that one single *E. coli* isolate may carry two CTX-M (−15 and − 27) variants capable of strongly hydrolyzing ceftazidime and belonging to two different groups of CTX-Ms (Group 1 and 9) is another example of concentration of restriction mechanisms.

Our findings further support the shift from *bla*_OXA-48-like_ variants toward *bla*_NDM-5_ among *E. coli* in hospital settings in Lebanon (12, 35). OXA-48-like enzymes were the most prevalent carbapenemases among Enterobacterales in Lebanon over the last decade while NDM-5 was uncommon in hospital and community settings (12, 40). In our study, the *bla*_NDM-5_ gene has become the predominant carbapenemase gene (14 of 26 *E. coli* isolates). The *bla*_OXA-244 gene_ (10/26) came in second place, while *bla*_OXA-48_ (1/33), and *bla*_OXA-181_ (1/33) genes are rare. OXA-244 producing *E. coli* isolates are increasingly described worldwide (41), and represents a threat to public health because of the difficulties in their detection using classical screening media based on carbapenems and temocillin (42). Indeed, our results confirmed that OXA-244-producing isolates have lower MICs to temocillin and carbapenems as compared to other CP-E, which results in the absence of growth on screening media and thus an underestimation and silent spread. Furthermore, these isolates were negative for the Carba NP test, and only the use of an ICA (here the NG-Test Carba5) revealed a positive signal for OXA-48-like carbapenemase, suggesting that both tests should be used together to maximize the chances of detecting all OXA-48-like carbapenemases. Additionally, the CR *K. pneumoniae* isolates harbored *bla*_OXA-48_ (3/5) and *bla*_NDM-1_ (2/5), while the *E. cloacae* and *E. hormaechei* isolates harbored *bla*_OXA-48_ and *bla*_NDM-1_, respectively.

MLST results demonstrated several distinct genetic backgrounds for the CR-E isolates, suggesting horizontal gene transfer of the carbapenemase gene carrying plasmids, particularly those carrying the *bla*_NDM-5_ gene, rather than a clonal spread of a single clone. Interestingly, we found that the carbapenemase genes were plasmid-borne (except in eight *bla*_OXA-244_-producing *E. coli*) and on different plasmid types (Table 4). Of the NDM-5-producing *E. coli* isolates, six and seven had the insertion of YRIN or YRIK in PBP3, respectively, which resulted in a significant increase of MICs to aztreonam/avibactam and cefiderocol as well as to other PBP3-targeting ß-lactams (34). Surprisingly, the *bla*_OXA-244_ gene was chromosomally encoded in 8 isolates, while it was plasmid-mediated in only two isolates belonging to ST69 and ST10. The chromosomal location of the *bla*_OXA-244_ gene was previously reported in ST38 and ST69 in France (43). Taken together, the occurrence of different resistance markers in diverse genetic backgrounds as well as plasmid types constitute a risk to patients, potentially highlighting a more severe problem and highlighting an urgent need to monitor and control the spread of resistance in hospitals in Lebanon.

The study identified various MLST types, including ST648, ST167, ST361, and ST405, in *bla*_NDM-5_-producing *E. coli*. These STs are recognized as high-risk global clones that contribute to the widespread dissemination of drug resistance determinants among *Enterobacterales* (44). For instance, ST648 is recognized as a major global ESBL-producing *E. coli* clone (45, 46), particularly associated with *bla*_CTX-M-15_, in humans, birds, and companion animals (47). Additionally, ST648 was identified in Lebanon among clinical CRE (48), including *bla*_NDM-5-_producing *E. coli* (49). Besides, ST167 has been linked to the global spread of *bla*_NDM_ in humans, animals, and food (50). ST405 has been detected in several countries, including the United States (51), Japan (52), and Lebanon (48), allowing the transmission of *bla*_CTX-M-15_ and *aac(6′)-Ib-cr* genes (53). Notably, both ST361 and ST648 have been reported among Syrian refugees in Lebanon (54), and ST90 was found in the effluent of Al-Qaa refugee camp (55). Additionally, we identified ST38 among *bla*_OXA-244_-producing *E. coli*, previously described in estuary water in Lebanon (56). Several European countries have reported increased dissemination of the *bla*_OXA-244_ gene (57, 58), with ST38 being the most common sequence type among *bla*_OXA-244_-producing *E. coli* isolates (59). Moreover, ST38 *bla*_OXA-48_-producing *E. coli* has also been found in fowls in Lebanon (60). As observed in our study, ST940 *E. coli* carrying the *bla*_OXA-181_ gene was previously reported at the American University of Beirut Medical Center (61). Among the *K. pneumoniae* isolates, there were five isolates belonging to four different sequence types. One of them, *K. quasipneumoniae* subsp. *similipneumoniae* ST1770, has been previously reported in hospital wastewater effluents in Japan (62), but had not been reported in Lebanon. Additionally, clinical *K. pneumoniae* isolates ST35 and ST45 have been documented in Lebanon (63, 64). ST37 has also been closely associated with ESBLs (65). Furthermore, the ST182 *bla*_NDM-1_ *E. hormaechei* isolate, previously reported in Lebanon (66), has been frequently isolated from clinical specimens in China, Mexico, the Czech Republic, and the United States (67).

Although there is a paramount need to monitor the spread of critical AMR strains in hospitals in Lebanon, these efforts are complicated by the unavailability of resources. Molecular typing methods such as whole genome sequencing are relatively time-consuming and expensive (32). Therefore, we evaluated the Bruker IR Biotyper for reliable detection of the relatedness and discrimination between strains. Our findings showed that Fourier Transform Infrared (FT-IR) technology is a powerful tool for strain typing, showing slightly better results to MLST and comparable results to WGS among CR-*E. coli* and *K. pneumoniae* isolates. Its advantages are summed up in the simple preparation of samples, ease of use, and low running costs. Together with its relatively high discriminatory power, the FT-IR seems to be a good tool for outbreaks real-time surveillance and infection control in clinical settings.

In conclusion, our study showed that in-depth studies are crucial to better understand the emergence and dissemination of drug-resistant determinants within and across healthcare institutions. Specifically, we highlighted an unprecedented diversity of ESBL- and CR-E determinants compared to other studies in Lebanon. Furthermore, the MLST and the associated plasmid types suggested that these determinants were circulating in diverse strains, complicating the control efforts and suggesting the need for evidence-based antimicrobial stewardship programs. In the distressing situation of Lebanon, the accessibility to novel antibiotic molecules remains a major concern, impacting public health. The economic crisis had a profound impact leading to significant challenges in antibiotics procurement and even more so to new molecules, which are unavailable and not yet routinely tested. Furthermore, our data support that the fight against MDR bacteria in LMICs, such as Lebanon, requires a comprehensive One Health approach because of the diffuse sources and factors that affect the spread of resistance in hospitals and the community. The latter corroborates our previous research that showed a wide reliance on and sometimes indiscriminate use of critically important antibiotics in healthcare settings, agriculture, and the community in Lebanon (8, 9). This approach and science-based interventions are urgently needed to control the spread of AMR in Lebanon, which represents an essential threat nationally and globally as AMR is known to spill across international borders.

## Data availability statement

The datasets presented in this study can be found in online repositories. The names of the repository/repositories and accession number(s) can be found at http://www.ncbi.nlm.nih.gov/bioproject and PRJNA973232.

## Ethics statement

The studies involving humans were approved by Azm Center/Lebanese University ethical committee and the Lebanese Ministry of Public Health (CE-EDST-1-2020). The studies were conducted in accordance with the local legislation and institutional requirements. The human samples used in this study were acquired from bacterial isolates collected based on susceptibility profiles from three hospitals. Written informed consent for participation was not required from the participants or the participants’ legal guardians/next of kin in accordance with the national legislation and institutional requirements.

## Author contributions

DD: Conceptualization, Formal analysis, Investigation, Writing – original draft, Writing – review & editing. SO: Supervision, Validation, Writing – review & editing. MR: Investigation, Writing – review & editing. IK: Investigation, Resources, Writing – review & editing. HM: Investigation, Writing – review & editing. AB: Formal analysis, Validation, Writing – review & editing. DG: Formal analysis, Investigation, Writing – review & editing. MH: Methodology, Writing – review & editing. FD: Formal analysis, Investigation, Writing – review & editing. MO: Conceptualization, Funding acquisition, Investigation, Methodology, Project administration, Validation, Writing – original draft, Writing – review & editing. TN: Conceptualization, Funding acquisition, Investigation, Methodology, Writing – original draft, Writing – review & editing, Project administration, Validation.

## Footnotes

^1^ https://www.eucast.org/clinical_breakpoints

^2^ https://www.sfm-microbiologie.org/wp-content/uploads/2021/12/CASFM_VET2021.pdf

^3^ https://cge.food.dtu.dk/services/ResFinder/

^4^ www.comparingpartitions.info
