# Supplementary material for: The emergence of carbapenemase-producing Enterobacterales in hospitals: a major challenge for a debilitated healthcare system in Lebanon
Source: Front Public Health. 2023 Nov 20;11:1290912. doi: 10.3389/fpubh.2023.1290912 (PMC10699444; doi:10.3389/fpubh.2023.1290912)
Supplement: Supplementary file 1 [file Data_Sheet_1.docx]

Supplemental Material

**1. Supplemental figures and tables**

**1.1. Supplemental figures**


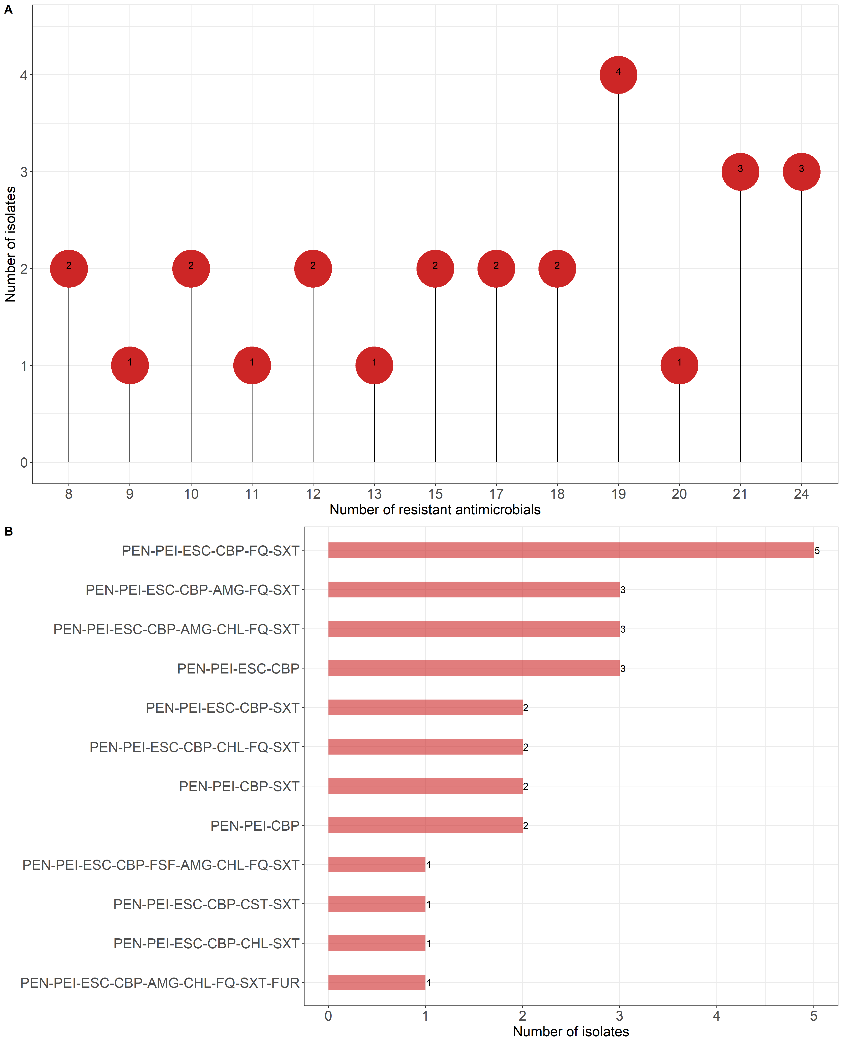


**Figure S1. Antimicrobial Susceptibility Patterns of Carbapenemase-Producing *Escherichia coli* (n=26) clinical isolates.** Distribution of resistance by number of antimicrobial agents (A) and susceptibility patterns of the isolates (B); Antimicrobial category abbreviations: PEN, penicillin; PEI, penicillin/β-lactamase inhibitor; ESC, extended-spectrum cephalosporins; CBP, carbapenems; AMG, aminoglycosides; CHL, chloramphenicol; TET, tetracycline; FQ, fluoroquinolones; SXT, trimethoprim-sulfamethoxazole; FUR, nitrofurantoin. This list excludes the tested antimicrobials in Table 2.


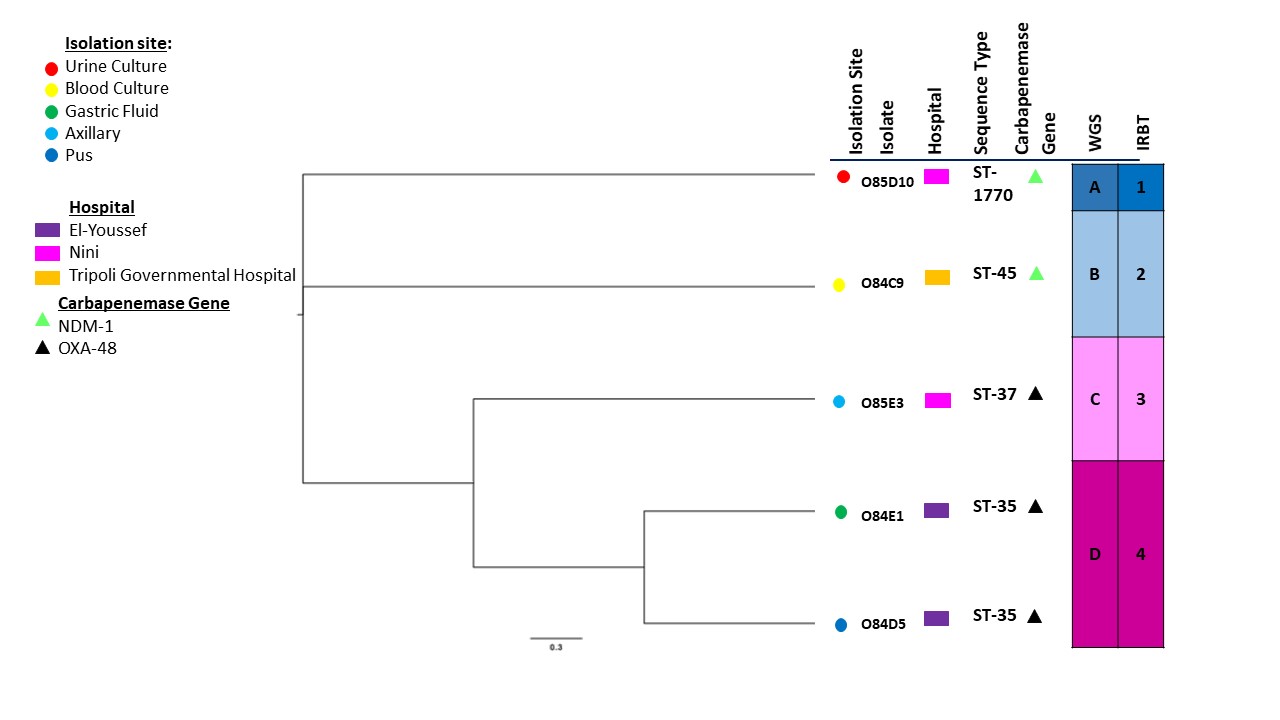


**Figure S2.** Phylogenetic tree of five carbapenemase-producing *Klebsiella pneumoniae*. A dendrogram was obtained by SNP‐based analysis, using CSI Phylogeny 1.4 available online at the center for genomic epidemiology-CGE (<http://www.genomicepidemiology.org/>)([1](#_ENREF_1)). The dendrogram was rooted with branch transform using FigTree software. IRBT and WGS types were assigned for each isolate and presented in different colors The STs, hospital, isolate ID, isolation site, and carbapenemase genes are also shown.


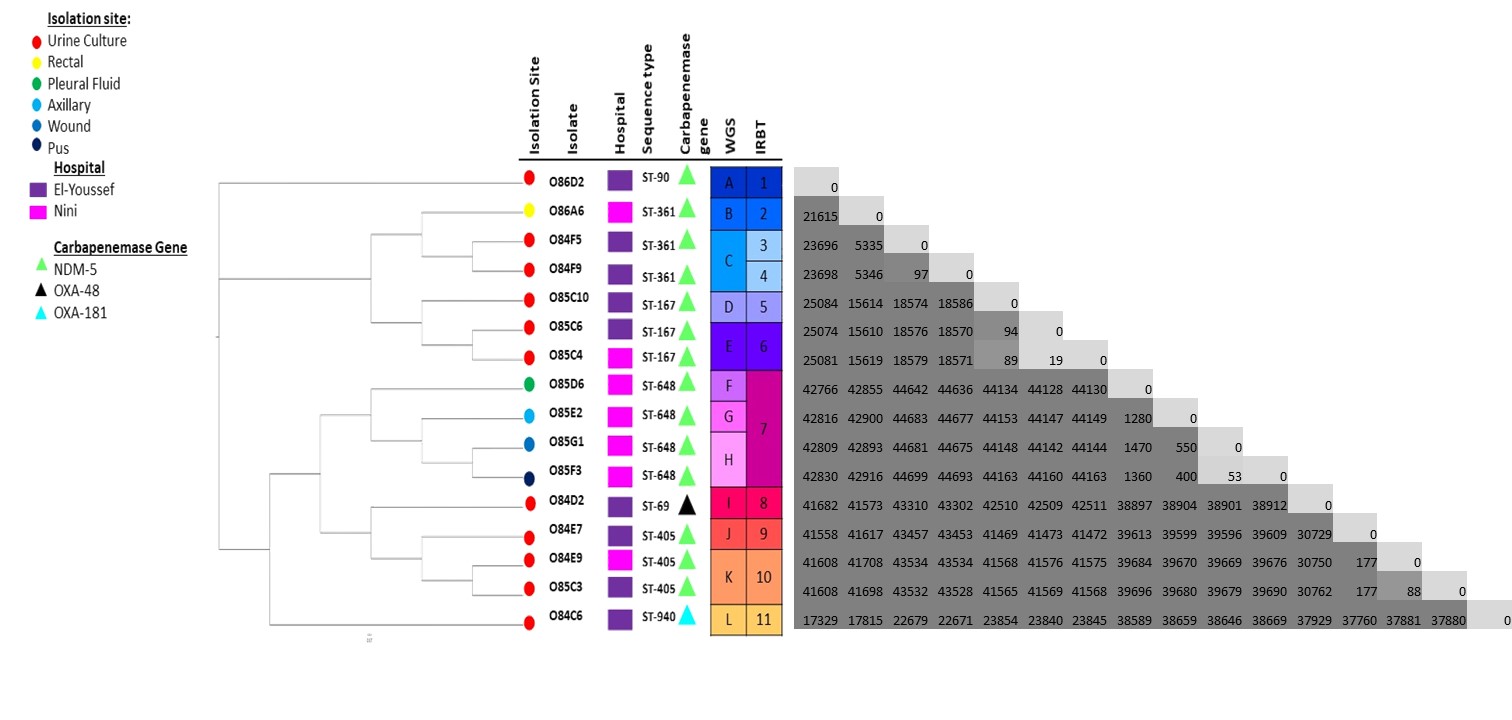


**Figure S3.** Phylogenetic tree of 16 carbapenemase-producing *Escherichia coli isolates*. A dendrogram was obtained by SNP‐based analysis, using CSI Phylogeny 1.4 available online at the center for genomic epidemiology-CGE (<http://www.genomicepidemiology.org/>) ([1](#_ENREF_1)).The dendrogram was rooted with branch transform using Figtree software. IRBT and WGS type was determined for each isolate and presented as different colors. The STs, hospital, isolate ID, isolation site, and carbapenemase genes are also shown.

**1. 2. Supplemental tables**

**Table S1.** Distribution of extended-spectrum β-lactamase genes carried by ESBL-producing *Enterobacterales* isolates.

| **Biochemical and Immunochromatographic tests** | **Species** | **Extended-spectrum β-lactamase gene** | **MLST type** |
| --- | --- | --- | --- |
| **β LACTA (+) / NG-Test CTX-M MULTI (+)** | *Escherichia coli* (n=43) | *bla*_CTX-M-15_ (n=40) | ND^1^ |
| (n=55) |  | *bla*_CTX-M-55_ (n=2) | ND |
|  |  | *bla*_CTX-M-3_ (n=1) | ND |
|  | *Klebsiella pneumoniae* (n=7) | *bla*_CTX-M-15_ (n=7) | ND |
|  | *Enterobacter* spp. (n=4) | *bla*_CTX-M-15_ (n=4) | ND |
|  | *Proteus mirabilis* (n=1) | *bla*_CTX-M-15_ (n=1) | ND |
| **β LACTA (+) / NG-Test CTX-M MULTI (-)** | *Escherichia. coli* O86A3 (n=1) | *bla*_SHV-12_ (n=1) | ST167 (Isolated in Nini Hospital) |
| (n=2) | *Klebsiella pneumoniae* O85A6 (n=1) | *bla*_SHV-187_ (n=1) | ST14 (Isolated in Nini Hospital) |

^1^ND, not determined.

**Table S2.** Comparison between disk diffusion (DD) and E-test (bioMérieux, Marcy-l’Étoile, France) results for ertapenem, imipenem, and meropenem, and broth microdilution method for temocillin. Major discordance (S/R and R/S) are shown in dark grey and minor discordance (S/I, I/S, R/I, and I/R) are shown in light grey. Results were interpreted according to the EUCAST guidelines ([2](#_ENREF_2)).

| **Isolate code** | **CarbaNP** | **NG-Test Carba5** | **β LACTA** | **NG-Test CTX-M MULTI** | **Ertapenem** | | **Imipenem** | | **Meropenem** | | **Temocillin** | |
| --- | --- | --- | --- | --- | --- | --- | --- | --- | --- | --- | --- | --- |
| ***Escherichia coli*** |  |  |  |  | DD (diameter mm) | MIC (mg/l) | DD (diameter mm) | MIC (mg/l) | DD(diameter mm) | MIC (mg/l) | DD (diameter mm) | MIC (mg/l) |
| O84C6 | + | OXA-like | + | - | R (15) | 0.5 | S (22) | 0.5 | S (22) | 0.75 | R (6) | 2048 |
| O84C10 | - | OXA-like | + | + | R (15) | 0.5 | S (33) | 0.25 | S (22) | 0.25 | R (10) | 64 |
| O84D2 | + | OXA-like | - | ND | R (19) | 0.25 | S (25) | 0.25 | S (23) | 0.125 | R (8) | 256 |
| O84D6 | - | OXA-like | + | + | R (6) | >32 | S (35) | 0.25 | S (22) | 0.25 | R (12) | 64 |
| O84E5 | - | OXA-like | - | ND | R (6) | 0.38 | S (25) | 4 | S (22) | 0.25 | R (12) | 16 |
| O84E7 | + | NDM | - | ND | R (6) | >32 | R (10) | 6 | R (6) | >32 | R (6) | 512 |
| O84E8 | - | OXA-like | - | ND | R (6) | 0.25 | S (35) | 0.25 | S (22) | 0.25 | R (15) | 16 |
| O84E9 | + | NDM | + | + | R(6) | >32 | R (12) | 3 | R (6) | >32 | R (6) | 1024 |
| O84F2 | - | OXA-like | + | + | R (10) | 0.75 | S (35) | 0.25 | S (22) | 0.25 | R (12) | 32 |
| O84F5 | + | NDM | + | - | R(6) | >32 | R (10) | 16 | R (6) | >32 | R (6) | 512 |
| O86D2 | + | NDM | + | + | R (8) | 4 | R (18) | 2 | R (12) | 3 | R (6) | 64 |
| O84F9 | + | NDM | - | ND | R (6) | >32 | R (10) | >32 | R (6) | >32 | R (6) | 1024 |
| O84G8 | - | OXA-like | - | ND | R (6) | 0.25 | S (35) | 0.25 | S (22) | 0.25 | R (15) | 8 |
| O85A1 | - | OXA-like | + | + | R (10) | 1 | S (35) | 0.25 | S (22) | 0.25 | R (15) | 32 |
| O85A3 | - | OXA-like | - | ND | R (12) | 0.5 | S (35) | 0.25 | S (23) | 0.125 | R (16) | 32 |
| O85C3 | + | NDM | + | + | R (6) | >32 | R (18) | 1 | R (8) | 8 | R (6) | 512 |
| O85C6 | + | NDM | + | + | R (8) | 6 | R (16) | 2 | R (13) | 1 | R (6) | 512 |
| O85C10 | + | NDM | + | + | R (8) | 3 | R (12) | 0.5 | R (17) | 0.75 | R (6) | 256 |
| O85D6 | + | NDM | - | ND | R (6) | >32 | R (8) | >32 | R (6) | >32 | R (6) | 512 |
| O85D8 | - | OXA-like | - | ND | R (10) | 0.75 | S (28) | 0.5 | S (22) | 0.75 | R (8) | 256 |
| O85E2 | + | NDM | - | ND | R (6) | >32 | R (6) | >32 | R (12) | >32 | R (6) | 2048 |
| O85F3 | + | NDM | - | ND | R (6) | >32 | R (12) | 12 | R (6) | 12 | R (6) | 256 |
| O85G1 | + | NDM | - | ND | R (6) | >32 | R (12) | 8 | R (6) | 16 | R (6) | 256 |
| O86A2 | - | OXA-like | - | ND | R (6) | 0.25 | S (32) | 0.25 | S (22) | 0.25 | R (14) | 16 |
| O85C4 | + | NDM | + | + | R(8) | 4 | R(12) | 2 | R(6) | 2 | R (6) | 256 |
| O86A6 | + | NDM | + | + | R (6) | >32 | R (16) | 2 | R (8) | 3 | R (6) | 32 |
| ***Klebsiella pneumoniae*** |  |  |  |  |  |  |  |  |  |  |  |  |
| O84C9 | + | NDM | + | + | R (8) | 6 | S (22) | 3 | R (10) | 3 | R (6) | 64 |
| O84D5 | + | OXA-like | + | - | R (10) | 3 | S (22) | 1 | R (18) | 1 | R (6) | 512 |
| O84E1 | + | OXA-like | + | + | R (20) | 0.5 | S (22) | 0.5 | S (22) | 0.5 | R (8) | 256 |
| O85D10 | + | NDM | + | + | R | 2 | S (22) | 0.5 | S (22) | 0.75 | R (6) | 32 |
| O85E3 | + | OXA-like | + | + | R(12) | 4 | S (22) | 0.5 | R (15) | 1 | R (6) | 1024 |
| ***Enterobacter* spp.** |  |  |  |  |  |  |  |  |  |  |  |  |
| O84D8 | + | NDM | + | - | R (6) | >32 | S (22) | 4 | R (6) | 12 | R (6) | 128 |
| O84F3 | + | OXA-like | + | - | R (1) | 6 | S (22) | 1 | R (15) | 1.5 | R (6) | 512 |

*S, Susceptible; R, Resistant; ND, Not determined.

**References**

1. Kaas RS, Leekitcharoenphon P, Aarestrup FM, Lund O. Solving the problem of comparing whole bacterial genomes across different sequencing platforms. PloS one. 2014;9(8):e104984.

2. EUCAST, European Society of Clinical Microbiology and Infectious Disease Guidelines. Recommandations 2023
