# Supplementary material for: The emergence of carbapenemase-producing Enterobacterales in hospitals: a major challenge for a debilitated healthcare system in Lebanon
Source: Front Public Health. 2023 Nov 20;11:1290912. doi: 10.3389/fpubh.2023.1290912 (PMC10699444; doi:10.3389/fpubh.2023.1290912)
Supplement: Supplementary file 3 [file Table_3.docx]

| Isolate code | MLST type | *bla*_NDM-1_ | *bla*_NDM-5_ | *bla*_OXA-48_ | *bla*_OXA-181_ | *bla*_OXA-244_ | *bla*_CTX-M-15_ | *bla*_CTX-M-27_ | *bla*_TEM-35_ | *bla*_TEM-1B_ | *blaSHV-110* | *bla*_SHV-12_ | *bla*_SHV-33_ | *blaSHV-26* | *bla*_OXA-1_ | *bla*_DHA-1_ | *bla*_ACT-16_ | *bla*_CMY-145_ | *bla*_OKP-B3_ | *aadA1* | *aadA2* | *aadA5* | *rmtB* | *rmtC* | *armA* | *aph(3’)-Ia* | *aph(3'’)-Ib* | *aac(3)-IIa* | *aph(6)-Id* | *tet(A)* | *tet(B)* | *qnrS1* | *qnrB1* | *qnrB4* | *qnrB19* | *aac(6’)-Ib-cr* | *sul1* | *sul2* | *sul3* | *dfrA1* | *dfrA12* | *dfrA17* | *dfrA14* | *fosA* | *fosA3* | *mphE* | *mph(A)* | *catA1* | *catB3* | *qacE* | *erm(42)* | *erm(B)* | *mdf(A)* | *sitABCD* | qepA | oqxA | oqxB |
| --- | --- | --- | --- | --- | --- | --- | --- | --- | --- | --- | --- | --- | --- | --- | --- | --- | --- | --- | --- | --- | --- | --- | --- | --- | --- | --- | --- | --- | --- | --- | --- | --- | --- | --- | --- | --- | --- | --- | --- | --- | --- | --- | --- | --- | --- | --- | --- | --- | --- | --- | --- | --- | --- | --- | --- | --- | --- |
| ***Escherichia coli*** | | | | | | | | | | | | | | | | | | | | | | | | | | | | | | | | | | | | | | | | | | | | | | | | | | | | | | | | | |
| O84D6** | ST10 |  |  |  |  |  |  |  |  |  |  |  |  |  |  |  |  |  |  |  |  |  |  |  |  |  |  |  |  |  |  |  |  |  |  |  |  |  |  |  |  |  |  |  |  |  |  |  |  |  |  |  |  | 99 |  |  |  |
| O85D8** | ST10 |  |  |  |  |  |  |  |  |  |  |  |  |  |  |  |  |  |  |  |  |  |  |  |  |  |  |  |  |  |  |  |  |  |  |  |  |  |  |  |  |  |  |  |  |  |  |  |  |  |  |  |  | 99 |  |  |  |
| O84F2** | ST38 |  |  |  |  |  |  |  |  |  |  |  |  |  |  |  |  |  |  |  |  |  |  |  |  |  |  |  |  |  |  |  |  |  |  |  |  |  |  |  |  |  |  |  |  |  |  |  |  |  |  |  |  |  |  |  |  |
| O84C10** | ST46 |  |  |  |  |  |  |  |  |  |  |  |  |  |  |  |  |  |  |  |  |  |  |  |  |  |  |  |  |  |  |  |  |  |  |  |  |  |  |  |  |  |  |  |  |  |  |  |  |  |  |  |  |  |  |  |  |
| O84D2** | ST69 |  |  |  |  |  |  |  |  |  |  |  |  |  |  |  |  |  |  |  |  |  |  |  |  |  |  |  |  |  |  |  |  |  |  |  |  |  |  |  |  |  |  |  |  |  |  |  |  |  |  | 99 | 99 |  |  |  |  |
| O84E5** | ST69 |  |  |  |  |  |  |  |  |  |  |  |  |  |  |  |  |  |  |  |  |  |  |  |  |  |  |  |  |  |  |  |  |  |  |  |  |  |  |  |  |  |  |  |  |  |  |  |  |  |  |  |  |  |  |  |  |
| O84E8** | ST69 |  |  |  |  |  |  |  |  |  |  |  |  |  |  |  |  |  |  |  |  |  |  |  |  |  |  |  |  |  |  |  |  |  |  |  |  |  |  |  |  |  |  |  |  |  |  |  |  |  |  |  |  |  |  |  |  |
| O84G8** | ST69 |  |  |  |  |  |  |  |  |  |  |  |  |  |  |  |  |  |  |  |  |  |  |  |  |  |  |  |  |  |  |  |  |  |  |  |  |  |  |  |  |  |  |  |  |  |  |  |  |  |  |  |  |  |  |  |  |
| O85A3* | ST69 |  |  |  |  |  |  |  |  |  |  |  |  |  |  |  |  |  |  |  |  |  |  |  |  |  |  |  |  |  |  |  |  |  |  |  |  |  |  |  |  |  |  |  |  |  |  |  |  |  |  |  |  |  |  |  |  |
| O86A2* | ST69 |  |  |  |  |  |  |  |  |  |  |  |  |  |  |  |  |  |  |  |  |  |  |  |  |  |  |  |  |  |  |  |  |  |  |  |  |  |  |  |  |  |  |  |  |  |  |  |  |  |  |  |  | 99 |  |  |  |
| O86D2** | ST90 |  |  |  |  |  |  |  |  |  |  |  |  |  |  |  |  |  |  |  |  |  |  |  |  |  |  |  |  |  |  |  |  |  |  |  |  |  |  |  |  |  |  |  |  |  |  |  |  |  | 100 |  |  |  |  |  |  |
| O85C6** | ST167 |  |  |  |  |  |  |  |  |  |  |  |  |  |  |  |  |  |  |  |  |  |  |  |  |  |  |  |  |  |  |  |  |  |  |  |  |  |  |  |  |  |  |  |  |  |  |  |  |  |  |  | 99 |  |  |  |  |
| O85C4* | ST167 |  |  |  |  |  |  |  |  |  |  |  |  |  |  |  |  |  |  |  |  |  |  |  |  |  |  |  |  |  |  |  |  |  |  |  |  |  |  |  |  |  |  |  |  |  |  |  |  |  |  |  |  |  |  |  |  |
| O85C10** | ST167 |  |  |  |  |  |  |  |  |  |  |  |  |  |  |  |  |  |  |  |  |  |  |  |  |  |  |  |  |  |  |  |  |  |  |  |  |  |  |  |  |  |  |  |  |  |  |  |  |  |  |  | 99 |  |  |  |  |
| O84F5** | ST361 |  |  |  |  |  |  |  |  |  |  |  |  |  | 100 |  |  |  |  | 99 |  |  |  |  |  |  |  |  |  |  |  |  |  |  |  |  |  |  |  |  |  |  |  |  |  |  |  | 100 |  |  |  |  | 99 | 99 | 99 |  |  |
| O84F9** | ST361 |  |  |  |  |  |  |  |  |  |  |  |  |  |  |  |  |  |  | 99 |  |  |  |  |  |  |  |  |  |  |  |  |  |  |  |  |  |  |  |  |  |  |  |  |  |  |  | 100 |  |  |  |  | 99 | 99 | 99 |  |  |
| O86A6* | ST361 |  |  |  |  |  |  |  |  |  |  |  |  |  |  |  |  |  |  |  |  |  |  |  |  |  |  |  |  |  |  |  |  |  |  |  |  |  |  |  |  |  |  |  |  |  |  |  |  |  |  | 99 | 99 | 99 |  |  |  |
| O84E7* | ST405 |  |  |  |  |  |  |  |  |  |  |  |  |  |  |  |  |  |  |  |  |  |  |  |  |  |  |  |  |  |  |  |  |  |  |  |  |  |  |  |  |  |  |  |  |  |  | 100 |  |  |  |  | 99 |  |  |  |  |
| O84E9* | ST405 |  |  |  |  |  |  |  |  |  |  |  |  |  |  |  |  |  |  |  |  |  |  |  |  |  |  |  |  |  |  |  |  |  |  |  |  |  |  |  |  |  |  |  |  |  |  |  |  |  |  |  | 99 |  |  |  |  |
| O85C3** | ST405 |  |  |  |  |  |  |  |  |  |  |  |  |  |  |  |  |  |  |  |  |  |  |  |  |  |  |  |  |  |  |  |  |  |  |  |  |  |  |  |  |  |  |  |  |  |  | 100 |  |  |  |  | 99 |  |  |  |  |
| O85E2* | ST648 |  |  |  |  |  |  |  |  |  |  |  |  |  |  |  |  |  |  |  |  |  |  |  |  |  |  |  |  |  |  |  |  |  |  |  |  |  |  |  |  |  |  |  |  |  |  |  |  |  |  | 99 | 99 |  |  |  |  |
| O85F3* | ST648 |  |  |  |  |  |  |  |  |  |  |  |  |  |  |  |  |  |  |  |  |  |  |  |  |  |  |  |  |  |  |  |  |  |  |  |  |  |  |  |  |  |  |  |  |  |  |  |  |  |  | 99 | 99 |  |  |  |  |
| O85G1* | ST648 |  |  |  |  |  |  |  |  |  |  |  |  |  |  |  |  |  |  |  |  |  |  |  |  |  |  |  |  |  |  |  |  |  |  |  |  |  |  |  |  |  |  |  |  |  |  |  |  |  |  | 99 | 99 |  |  |  |  |
| O85D6* | ST648 |  |  |  |  |  |  |  |  |  |  |  |  |  |  |  |  |  |  |  |  |  |  |  |  |  |  |  |  |  |  |  |  |  |  |  |  |  |  |  |  |  |  |  |  |  |  |  |  |  |  | 99 | 99 |  |  |  |  |
| O85A1** | ST8881 |  |  |  |  |  |  |  |  |  |  |  |  |  | 100 |  |  |  |  |  |  |  |  |  |  |  |  |  |  |  |  |  |  |  |  |  |  |  |  |  |  |  |  |  |  |  |  |  |  |  |  | 99 |  |  |  |  |  |
| O84C6** | ST940 |  |  |  |  |  |  |  |  |  |  |  |  |  |  |  |  |  |  |  |  |  |  |  |  |  |  |  |  |  |  |  |  |  |  |  |  |  |  |  |  |  |  |  |  |  |  |  |  |  |  |  | 99 |  |  |  |  |
| ***Klebsiella pneumoniae*** | | | | | | | | | | | | | | | | | | | | | | | | | | | | | | | | | | | | | | | | | | | | | | | | | | | | | | | | | |
| O84E1** | ST35 |  |  |  |  |  |  |  |  |  |  |  |  |  |  |  |  |  |  |  |  |  |  |  |  |  |  |  |  |  |  |  |  |  |  |  |  |  |  |  |  |  |  | 99 |  |  |  |  |  |  |  |  |  |  |  | 99 | 99 |
| O84D5** | ST35 |  |  |  |  |  |  |  |  |  |  |  |  |  |  |  |  |  |  |  |  |  |  |  |  |  |  |  |  |  |  |  |  |  |  |  |  |  |  |  |  |  |  |  |  |  |  | 100 |  |  |  |  |  |  |  |  |  |
| O85E3* | ST37 |  |  |  |  |  |  |  |  |  | 99 |  |  |  |  |  |  |  |  |  |  |  |  |  |  |  |  |  |  |  |  |  |  |  |  |  |  |  |  |  |  |  |  | 98 |  |  |  |  |  |  |  | 99 |  |  |  | 100 |  |
| O84C9*** | ST45 |  |  |  |  |  |  |  |  |  |  |  |  |  |  |  |  |  |  |  |  |  |  |  |  |  |  |  |  |  |  |  |  |  |  |  |  |  |  |  |  |  |  |  |  |  |  |  |  |  |  |  |  |  |  | 99 |  |
| O85D10* | ST1770 |  |  |  |  |  |  |  |  |  |  |  |  |  |  |  |  |  |  |  |  |  |  |  |  |  |  |  |  |  |  |  |  |  |  |  |  |  |  |  |  |  |  |  |  |  |  |  |  |  |  |  |  |  |  |  |  |
| ***Enterobacter* spp.** | | | | | | | | | | | | | | | | | | | | | | | | | | | | | | | | | | | | | | | | | | | | | | | | | | | | | | | | | |
| O84D8* | ST182 |  |  |  |  |  |  |  |  |  |  |  |  |  |  |  |  |  |  |  |  |  |  |  |  |  |  |  |  |  |  |  |  |  |  |  | 99 |  |  |  |  |  |  | 96 |  |  |  |  |  |  |  |  |  |  |  |  |  |
| O84F3** | ST1006 |  |  |  |  |  |  |  |  |  |  |  |  |  |  |  |  |  |  |  |  |  |  |  |  |  |  |  |  |  |  |  |  |  |  |  |  |  |  |  |  |  |  | 97 |  |  |  |  |  |  |  |  |  |  |  |  |  |

**Table S3.** Species, MLST and Resistome of the 33 carbapenemase-producing Enterobacterales clinical isolates. Species, Sequence types and resistance genes were analyzed using KmerFinder 3.2, MLST 2.0 and ResFinder 4.1 tools available online at the Center for Genomic Epidemiology-CGE (<http://www.genomicepidemiology.org/>). Acquired antimicrobial resistance genes were detected using 95% identity as cut-off.
